# Development of a structured tracking system to improve retention in a birth cohort in rural Ecuador

**DOI:** 10.1080/16549716.2025.2569207

**Published:** 2025-10-14

**Authors:** Stephanie Montenegro, Alexis J. Handal, Fadya Orozco

**Affiliations:** a Centro de Transferencias y Desarrollo de Tecnologías CTT-USFQ, Universidad San Francisco de Quito USFQ, Quito, Ecuador; b Department of Epidemiology, University of Michigan School of Public Health, Ann Arbor, MI, USA

**Keywords:** developing countries, follow-up studies, participant tracking, home visits, partial refusals

## Abstract

Retention in birth cohort studies varies globally, with rates of over 80% in high-income countries and 30%–75% in low- and middle-income settings due to socioeconomic vulnerability, geographic dispersion, and infrastructural barriers. Despite the importance of participant retention, evidence on structured tracking systems contextualized to these settings remains limited. This paper describes the design, adaptations, and outcomes of a participant tracking system developed for the Study of Environmental Exposure of Mothers and Infants Impacted by Large-Scale Agriculture, a community-based birth cohort that enrolled 409 pregnant participants in a floriculture-intensive rural area of Ecuador. In this setting, retention was challenged by informal employment, variable phone access, and high mobility. The tracking system was implemented from pregnancy through 18  months postpartum, including two final follow-up waves conducted at 12 and 18  months. It comprised a predefined follow-up schedule, a team with defined roles, a real-time digital planner for recording contact attempts, participation status, and activity completion, and a five-tier participation classification. Tracking was conducted through office visits, phone calls, and home visits. Flexible protocols and targeted outreach addressed pandemic-related disruptions and irregular work schedules. Overall retention was 77%, with 84% and 75% at 12 and 18  months, respectively. Partial refusals (27%) were primarily linked to mobility and work demands, while 94 participants fully discontinued. Home visits enabled completion for 154 participants, with an 88% success rate. This experience illustrates how context-adapted tracking systems can sustain high retention in rural settings, offering a scalable model to inform global health research in underserved populations.

## Paper Context

- **Main Findings:** A context-adapted tracking system enabled high participant retention in a rural birth cohort, overcoming logistical, social, and contextual barriers in a low-resource setting.
- **Added Knowledge:** This study contributes new knowledge by documenting the design and field implementation of a context-adapted tracking system, offering an operational model beyond isolated retention strategies.
- **Global health impact for policy and action:** It supports global health efforts to strengthen long-term data quality through practical, scalable retention strategies in community-based research.

## Background

Retention is critical to the success of longitudinal birth cohort studies. Globally, retention rates vary substantially, with cohorts in high-income countries (HICs) reporting rates above 80% over extended follow-up periods, while those in low- and middle-income countries (LMICs), including sub-Saharan Africa, report values as low as 30% [1–4]. These differences reflect economic disparities and complex operational challenges, such as population mobility, informal employment, institutional mistrust, and infrastructural barriers [1,2,4].

Although various retention strategies have been documented in HICs, including digital reminders, integration with electronic health records, robust infrastructure, and established physical address systems that facilitate participant location, community-based cohorts in LMICs frequently rely on more labor-intensive approaches due to informal housing, limited internet access, and lower smartphone ownership [2,5]. There is limited recent evidence describing comprehensive tracking systems in LMICs, especially in rural or resource-constrained contexts. Most studies focused on isolated practices, without detailing operational frameworks that can be replicated across multiple waves of follow-up.

The Study of Environmental Exposure of Mothers and Infants Impacted by Large-Scale Agriculture (SEMILLA), a community-based birth cohort in the rural highlands of Ecuador, provides a suitable context to examine the design and field implementation of tracking systems for participant retention. The setting was characterized by high population mobility, substantial socioeconomic vulnerability, and a predominantly female workforce employment in floriculture. Structural challenges included limited health infrastructure, high mobility, informal employment, and inconsistent phone access, barriers for long-term follow-up in a low-resource environment. Nationally, 2024 statistics indicate that 61.3% of the population had an active mobile phone and 57.7% had smartphones, with even lower access in non-urban areas (47.4% vs 42.0%); 48.1% of rural households had internet access [6]. These gaps highlight the disadvantages of relying on mobile technologies for follow-up and differentiate our context from other LMICs with broader coverage. The cohort design and multiple follow-up visits across pregnancy, birth, and infancy allowed close observation of retention patterns over time.

We describe the design, adaptation, and field implementation of a participant tracking system in the SEMILLA study, providing practical guidance for improving retention rates and ensuring high data quality in community-based cohort studies conducted in underserved settings.

## Context and study setting

The SEMILLA study was conducted in Cayambe and Pedro Moncayo, Ecuador. Between October 2019 and April 2022, 409 pregnant women (8–20  weeks of gestation) were recruited from the general population to examine prenatal environmental exposures and child development. Data was collected during prenatal, birth, and infancy follow-up visits extending through October 2023. All participants signed an informed consent of detailed follow-up frequency and provided an alternative contact information. The study protocol has been described in detail previously [7].

In response to the COVID-19 pandemic and national protests, the protocol was revised in 2021, resulting in two final follow-up waves based on enrolment date: participants enrolled before 30 September 2021 were scheduled for an 18-month follow-up (Final Wave 2 [FW2]), and those recruited thereafter for a 12-month follow-up (Final Wave 1 [FW1]). Both schemes had similar study objectives, procedures, and types of information collected but different lengths of follow-up. Since retention is directly influenced by the frequency and duration of scheduled visits, distinguishing between the two Final Waves is important for interpreting participation patterns. The FW1/FW2 naming convention was retained to maintain consistency across study documentation and systems.

A structured tracking system was progressively developed and strengthened in response to operational challenges that emerged throughout the study, including the coronavirus disease (COVID-19) pandemic, national protests, and increasing participant mobility.

## Structure and operational design of the tracking system

The SEMILLA tracking system, developed specifically for the SEMILLA Study and adapted to its rural context was conceptualized as an operational framework composed of four interdependent components: (1) a predefined follow-up schedule with clear collection windows that included core assessment activities interviews, anthropometric measurements, and neurodevelopmental evaluations; (2) a dedicated field team; (3) a digital Tracking Planner to support real-time coordination and documentation; and (4) a five-tier participant classification system to monitor retention status and guide retention strategies.

These components shaped a flexible system that allowed timely adaptation to contextual challenges and made the SEMILLA experience unique among LMIC birth cohorts.

### Follow-up schedule and collection windows

The follow-up schedule was structured into *pregnancy*, *birth*, and *infancy*, to align fieldwork with key stages of maternal and child development. The baseline visit at enrollment was excluded from the follow-up schedule. Scheduled visits are summarized in Table 1.

**Table 1.** Scheduled follow-up visits by study period and final wave.

Study Period	Visit	FW1 early enrollment (8–14 wk)	FW1 late enrollment (15–20 wk)	FW2 early enrollment (8–14 wk)	FW2 late enrollment (15–20 wk)
Pregnancy	20 weeks	✓	—	✓	—
	32 weeks	✓	✓	✓	✓
Birth	0–14 days after birth	✓	✓	✓	✓
Infancy	3 months	✓	✓	✓	✓
	6 months	✓	✓	✓	✓
	9 months	✓	✓	✓	✓
	12 months	✓	✓	✓	✓
	15 months	—	—	✓	✓
	18 months	—	—	✓	✓

Participants were grouped by gestational age at enrollment (8–14 or 15–20 weeks) and assigned to FW1 or FW2. ✓: visit was scheduled for that group; —: not applicable. Collection windows applied to each visit. wk, weeks.

Participants were grouped by gestational age at enrollment (8–14 or 15–20  weeks) and assigned to FW1 or FW2. ✓: visit was scheduled; —: not applicable. Collection windows applied to each visit. wk, week.

Data collection windows were defined based on gestational or infant age. Pregnancy visits were conducted during the target gestational week, with up to six additional days. For example, a participant enrolled at 20  weeks could be followed for up to 6  days (until 20  weeks and 6  days), while one enrolled at 20  weeks and 3  days had only 3  days remaining to stay within the window. The birth visit was scheduled within the first 14  days postpartum. During infancy, each scheduled visit had a ≤20-day window per visit, allowing logistical flexibility while maintaining temporal consistency across visits.

**Figure 1.**
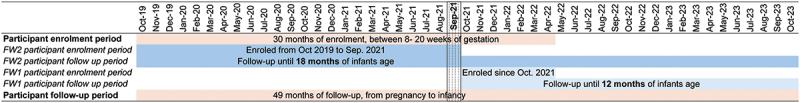
Timeline of recruitment and follow-up periods by final wave in the SEMILLA study. Recruitment occurred over 30 months (October 2019 to April 2022). The shaded area marks September 2021, when a protocol modification was implemented. Participants enrolled until 30 September 2021, were followed up to 18 months postpartum (final wave 2), while those enrolled from 1 October 2021, were followed up to 12 months postpartum (final wave 1). Colored bars represent enrolment and follow-up periods for each group. The operational period lasted 49 months, from the enrolment of the first participant to the last follow-up visit. This reflects the overall study timeline and does not represent individual participant follow-up duration, which varied according to gestational age at enrolment and assigned follow-up group.

Figure 1 provides a visual summary of recruitment and follow-up periods.

### Scheduled follow-up visits: core activities and visit-specific assessments

Each follow-up visit generally consisted of: (1) administration of a main questionnaire addressing sociodemographic characteristics and exposure-related risk factors; (2) application of standardized scales at selected time points to assess maternal depression, stress, and breastfeeding practices; (3) collection of biological samples; and (4) age-specific evaluations of infant growth and neurodevelopment.

These activities were tailored to the study period and developmental stage of the child. Pregnancy visits involved maternal anthropometry and urine collection for exposure assessment. Birth visits involved neonatal anthropometry and blood sampling for mother and infant; a newborn venous blood sample was used to assess thyroid hormone levels. Infancy visits included anthropometric measurements, neurodevelopmental assessments, and visual acuity screening [7]. Pregnancy visits were typically completed in a single 2-h session, while birth and infancy visits were conducted over 2 days and required approximately three to 4 h in total.

Core assessment activities were prioritized for their relevance to study outcomes and used to define participation status across study periods (see Table 2).

**Table 2.** Core assessment activities used to classify participant status by study period in the SEMILLA study.

Assessment Activity	Study periods		
	Pregnancy	Birth	Infancy
Main questionnaire (sociodemographic, risk factors)	✓	✓	✓
Biological samples	Urine, blood (mother)	Blood (newborn)	—
Infant anthropometric evaluations	—	✓	✓
Infant neurodevelopmental assessments	—	—	✓

Core assessment activities were selected based on their alignment with the study’s primary objective, evaluating the relationship between prenatal exposure to ethylene thiourea (ETU) and maternal and infant thyroid function and neurodevelopment. ✓: scheduled in that period; —: not applicable.

### Tracking team and roles

This team included a tracker, two interviewers, and a technical coordinator.

The tracker managed participant contact information, supported retention efforts, and conducted home visits when participants could not be reached after multiple attempts, with prior verbal agreement to respect autonomy. Home visits were integrated into routine fieldwork to complete assessments, address concerns, and confirm willingness to continue.

Interviewers conducted scheduled assessment activities, recorded follow-up visit outcomes, and coordinated with the tracker on contact difficulties, rescheduling, or participant concerns. The technical coordinator reviewed the Tracking Planner weekly and guided operational adjustments with the Local Principal Investigator (FO).

The tracking team remained consistent throughout the study, with members who had at least secondary-level education and backgrounds in health or social fields. Their continuity and shared skills such as teamwork, interpersonal communication, early detection of participant needs, protocol adherence, and accurate record-keeping contributed to retention.

Each follow-up interview lasted approximately 2 h per participant, with up three participants per day (≈120 h per month). This represented 75% of their standard workload and ≈488 USD of their monthly salary. Comparable estimates were calculated for the tracker, while costs for the technical coordinator and the local principal investigator were excluded.

### The Tracking Planner

The digital planner was developed using Microsoft Excel and hosted on Google Drive, to coordinate follow-up activities in real time and maintain updated participant status information.

The *Tracking Planner* consisted of the *follow-up planner*, which tracked scheduled follow-up visits and outcomes, and the *participant contact data*, which recorded all relevant details and communication history (Table 3). A detailed overview of the data variables included in the Tracking Planner is provided in Table 3.

**Table 3.** Data variables included in the tracking Planner sheets.

Follow-up Planner	Participant Contact Data
*Participant identifier*	*Participant identifier*
*Enrollment date*	*Primary phone number*
*Collection windows start/end dates*	*Secondary phone number*
*Participant status classification*	*Preferred contact times*
*Reason for missed visit*	*Georeferenced address*
	*Comments on contact attempts*

Variables in the follow-up planner and participant contact data sheet.

The tool was reviewed continuously by the tracking team to monitor each participant’s follow-up status and detect any early signs of disengagement.

It facilitated coordination across follow-up points, supported timely field decisions, and ensured consistency in retention efforts. The Planner was implemented from the study‘s inception, requiring close coordination under tight operational constraints.

### Tracking process and participant status classification

**Figure 2.**
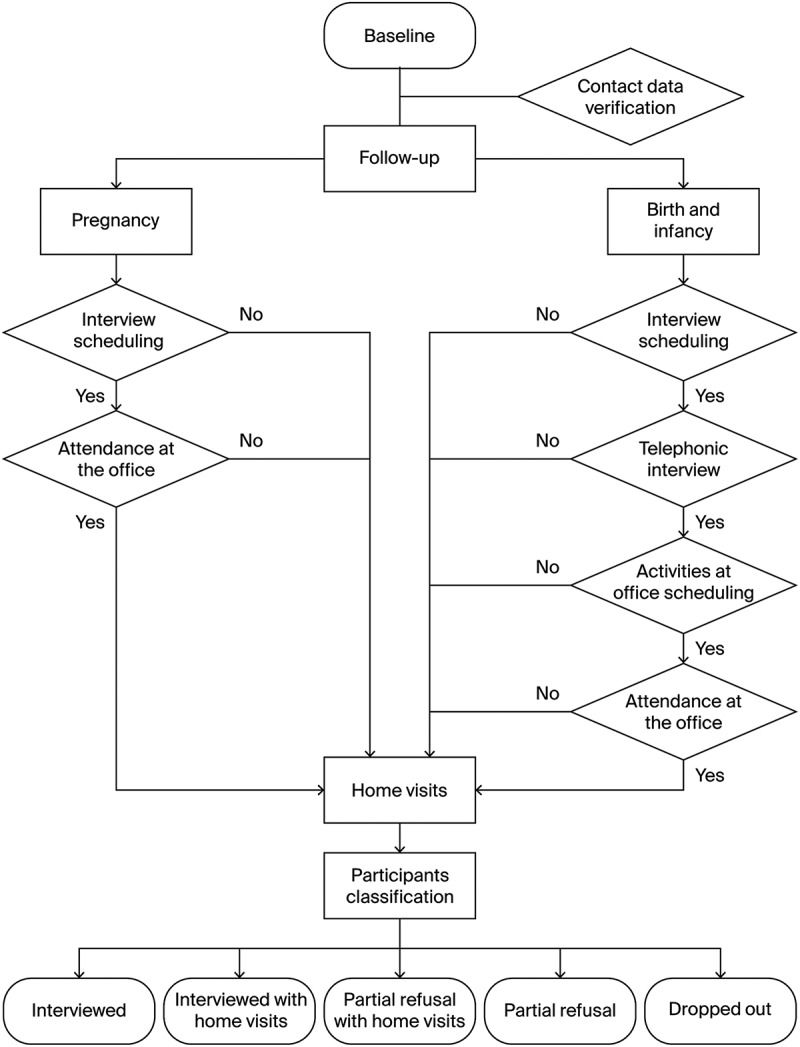
Tracking and classification flowchart used in the SEMILLA study. Key decision points and retention criteria used to assign participant status across follow-up visits are shown. Adapted from a figure originally presented in a poster at the ISEE 2024, Santiago, Chile. Reproduced with permission from the authors.

The tracking process was designed to monitor retention at each follow-up visit. After the baseline visit, conducted during pregnancy at the study office, the staff scheduled home visits to collect urine samples and verify contact information, starting the tracking process (see Figure 2).

Contact strategies directly informed classification. Participants were invited to attend in-office evaluations. During the birth period (0–14  days postpartum), interviews and in-person assessments were conducted by phone and at the study office, respectively. During infancy, interviews were conducted by phone, while neurodevelopmental and anthropometric assessments were done at the study site. After three failed contact attempts, a home visit was conducted to complete pending activities before assigning classification.

After each follow-up visit, participants were assigned to one of the five retention categories.

- Out-migration from the study area
- Disallowance of newborn venous blood sampling, despite initial consent. Essential for assessing thyroid hormone levels, a primary study outcome
- Abortion or stillbirth
- Voluntary withdrawal from the study
- Classification as ‘partial refusal’ in two consecutive scheduled follow-up visits, as this pattern resulted in the loss of consecutive follow-up data necessary for longitudinal analyses. This criterion was used to identify participants with sustained disengagement.

1. **Interviewed**: Completed core activities for the visit.
2. **Interviewed with home visits**: Completed core activities after receiving at least one home visit.
3. **Partial refusal** Declined one or more core activities within a visit but maintained their willingness to continue, as indicated by in-person or phone contact (adapted from Del Brutto et al. [8]).
4. **Partial refusal with home visits**: Initially missed or declined core activities, received a home visit to facilitate completion, and still declined one or more core activities yet remained willing to continue on future visits.
5. **Dropped out**: Defined by any of the following:
  - Out-migration from the study area
  - Disallowance of newborn venous blood sampling, despite initial consent. Essential for assessing thyroid hormone levels, a primary study outcome
  - Abortion or stillbirth
  - Voluntary withdrawal from the study
  - Classification as ‘partial refusal’ in two consecutive scheduled follow-up visits, as this pattern resulted in the loss of consecutive follow-up data necessary for longitudinal analyses. This criterion was used to identify participants with sustained disengagement.

Classification decisions were documented in the Tracking Planner and reviewed by the field team and the local principal investigator (FO) for consistency. Procedures respected participant autonomy; refusals were recorded without penalty, and adaptations, such as home visits, were offered to reduce participant burden and support voluntary engagement (Figure 2).

## Adaptations to operational design and implementation insights

The SEMILLA tracking system was implemented in a rural, dispersed, and socioeconomically vulnerable population requiring adaptations to address limited infrastructure, high mobility, and contextual disruptions. While ethnicity data were collected as part of the broader SEMILLA study, they were not used to guide tracking system design or field procedures. The system incorporated flexible, participant-centered strategies [2,5] across its four operational components, which served as a framework guiding its design and aligning with the study’s objective of evaluating the impact of prenatal ETU exposure on thyroid function and neurodevelopment. Core assessment activities were selected and applied across follow-up waves to determine participant status.

In April 2022, following a decline in pregnancy follow-up attendance observed during three-month infant visits, home visits were formally established as a retention strategy.

Real-time coordination was facilitated using the Tracking Planner. Weekly reviews by the technical coordinator supported timely decisions and field adjustments. Its low cost and adaptability made it suitable for resource-limited settings.

Participation was classified using a five-tier system which allowed for more nuanced tracking and tailored retention strategies such as rescheduling or home visits. Indicators like partial refusal frequency and home visit success rates were used to monitor implementation performance.

## Operational data analysis

Descriptive data analyses were conducted using Excel. The participants assigned to each classification category were counted at each follow-up point. Retained participants were those classified as interviewed, interviewed with home visits, partial refusal, or partial refusal with home visits. Retention rates were calculated using complementary measures: *overall retention* (participants retained at the end of follow-up divided by baseline cohort, *N* = 409) and *Final Wave retention* (12 and 18 months) calculated with both wave-specific denominators (participants enrolled in each wave) and the total cohort size (*N* = 409). We analyzed associations between retention status and selected baseline socio-demographic variables. These included: (1) *maternal age*; (2) *self-identified ethnicity* (Indigenous, Mestiza, Afro-Ecuadorian, White); (3) *marital status* (married, cohabiting, separated, divorced, widowed, single); (4) *years of schooling*, measured as total years completed; (5) *employment status* (employed vs. not employed); and (6) *employment secto*r (floriculture/agriculture, other sectors, or none). We created a dichotomous variable ‘Retained Participant’ (1 = yes, 0 = no) and calculated frequencies (%) for the categorical variables. Associations were tested using the Pearson‘s χ^2^ test or Fisher‘s Exact Test when expected cell counts were <5, while the Student’s *t* test was used for continuous variables.

Additional indicators, including: the percentage of partial refusals by reason (grouped into contextual and individual factors); the success rate of home visits (participants interviewed with home visits/total home visits); and the total cost of home visits, based on monthly budget records, described the tracking system’s functioning. To assess how reasons for partial refusal varied over time, refusal events were recorded at each follow-up visit and analyzed by wave, rather than by participant, as individuals could report different reasons at different points. Dropouts at each follow-up stage were also monitored to understand attrition patterns. Although some general reasons were documented, a detailed analysis will be presented in a separate manuscript focused on attrition in the SEMILLA cohort.

### Ethical considerations

The SEMILLA study was conducted in accordance with the ethical principles of the Declaration of Helsinki. Full details of ethical approval are provided in the ‘Ethics and consent’ section.

## Operational outcomes

Participants in the SEMILLA study were predominantly Spanish-speaking mestizo women, aged ≥18 years, who were working for income, and recruited from the general population.

### Participant retention and follow-up outcomes

**Figure 3.**
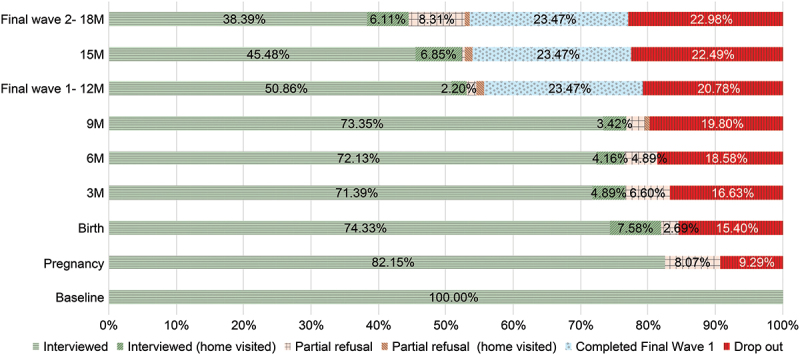
Retention rates across scheduled follow-up visits, including final waves the figure shows participant status at each follow-up visit, expressed as proportions of the baseline cohort (*N* = 409). The lower proportions observed at 15 and 18 months reflect the planned completion of follow-up for participants assigned to the 12-month final wave, rather than attrition. Categories include completed interviews (at the study office or via home visits), partial refusals (on-site and home visited), and cumulative drop-out. Adapted from a figure originally presented at the 2024 ISEE conference, Santiago, Chile. Reproduced with permission from the authors.

Retention rates varied across follow-up points. The number of participants classified as ‘interviewed’ remained relatively stable during early infancy, particularly at the three- (*n* = 292), six- (*n* = 295), and nine-month (*n* = 300) follow-up points (Figure 3).

Of the 115 participants enrolled in FW1, 96 concluded their participation at the 12-month visit as planned, yielding a retention rate of 84% (96/115). In FW2, 219 of 294 participants remained in the study through the 18-month visit, corresponding to a 75% retention rate (219/294). When expressed relative to the original cohort size (*N* = 409), retention rates were 23% and 54% for FW1 and FW2, respectively. This resulted in an overall retention rate of 77% (315/409).

Overall 94 participants dropped out, most during pregnancy (*n* = 39), followed by birth (*n* = 23). In subsequent visits, drop-out numbers ranged from two to eight participants (see Figure 3). As noted, 96 participants exited the study at the 12-month visit according to the protocol, leading to a reduction of the number of participants by approximately 30% between the 12- and 15-month visits.

General reasons for drop-out, such as relocation or communication barriers, were documented during tracking. A detailed analysis of these attrition patterns will be presented in a forthcoming manuscript focused on the SEMILLA cohort.

### Partial refusals: reasons and patterns

**Figure 4.**
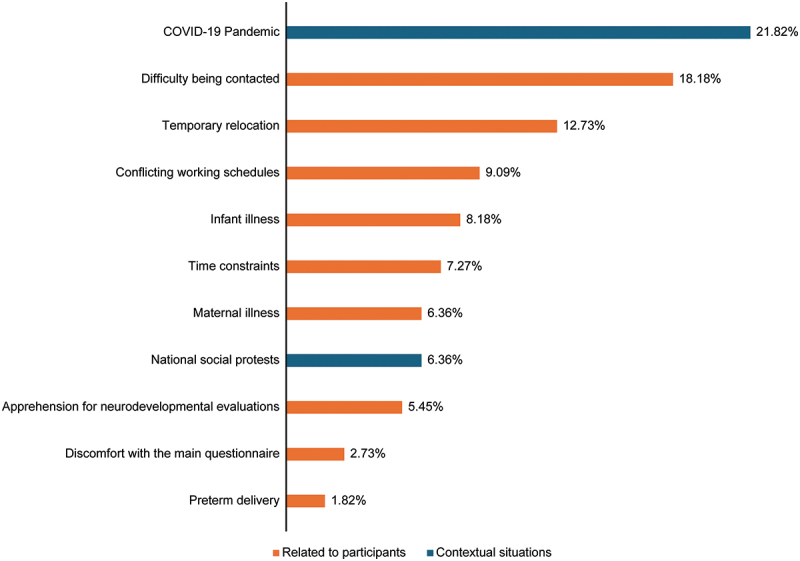
Contextual and participant-related reasons for partial refusals in the SEMILLA study. Distribution of reported reasons for partial refusals, grouped into participant-related and contextual factors based on field team classifications used during data collection and monitoring. Adapted from a version originally presented at the 2024 ISEE conference, Santiago, Chile. Reproduced with permission from the authors.

Overall 110 participants (27%) met all the criteria for partial refusal. Most cases (*n* = 93) occurred at a single follow-up point, while 17 were recorded in two or more non-consecutive visits. In total, 169 partial refusal events were recorded across follow-up waves, as some participants contributed more than once (Figure 4).

Individual factors included: difficulty being contacted, temporary relocation, conflicting work schedules, infant, or maternal illness, and time constraints. Additional reasons included apprehension about neurodevelopmental evaluation and discomfort with the main questionnaire. A few cases were related to preterm delivery. Among contextual factors, the COVID-19 pandemic was the most frequently reported reason, followed by national socialprotests.

Refusal reasons varied by time point. COVID-19-related refusals were most frequent at 18  months (23 events), during pregnancy (17), at birth (12), at 3  months (10), and at 6  months (7). Difficulty being contacted was reported most often at 12  months (six events), followed by 3 and 18 months (five each), and at 6 and 9 months (four each). Temporary relocation peaked at 6 months (five events). Discomfort with the main questionnaire was primarily reported at 12 months (two events), while aversion to neurodevelopment evaluation was noted at 18 months (four events).

### Impact of home visit strategy

Of the 176 participants who received at least one home visit, 154 completed their scheduled activities, yielding an 88% success rate. The home visit strategy required an estimated transportation investment of USD 2,500, with an average cost of USD 14.20 per visit.

Home visits contributed to participant engagement across all follow-up points. They increased the number of ‘interviewed’ participants during the birth follow-up point (*n* = 31) and in the later 15-month (*n* = 28) and 18-month (*n* = 25) follow-up points. At earlier follow-up points, they also supported additional interviews at 3  months (*n*  =  20), 6  months (*n*  =  17), 9  months (*n*  =  14), and 12  months (*n*  =  18) facilitating consistent classification across follow-up points in the Tracking Planner.

Home visits enabled in-person contact with otherwise unreachable participants across follow-up points, notably during the birth, 15-month, and 18-month visits.

### Sociodemographic factors associated with retention

Only maternal employment status (*p*  =  0.017) and self-identified ethnicity (*p*  =  0.007) were significantly associated with retention. Indigenous women showed higher retention (86.4%) compared to Mestiza women (74.8%). Although all Afro-Ecuadorian participants remained in the study (100%) and most White participants were lost to follow-up (75.0%), however, these variables were not significantly associated with retention. No other variables showed significant associations with retention status.

## Discussion

This report describes the operational design, contextual adaptations, and outcomes of a participant tracking system for a rural birth cohort in Ecuador. The system combines a predefined follow-up schedule, defined field team roles, a digital coordination tool, and a five-tier participation classification to support retention.

Contextual adaptations, including home visits, flexible scheduling, and multi-modal contact strategies, addressed barriers such as pandemic disruptions, irregular work hours, and participant mobility. These components sustained engagement despite structural and logistical challenges.

Retention rate reached 84% at 12  months and 75% at 18  months, comparable to the MINA-Brazil [9] and ISA-Costa Rica studies [10], and supported by a trained local team, real-time tracking, and systematic participation classification [11,12]. Similar LMIC cohorts, like the one in Vientiane in Lao People’s Democratic Republic [13], show that combining structured monitoring with local capacity sustains high retention.

The five-tier classification system helped monitor engagement and guide follow-up actions including full participation, partial refusals, and drop-outs. Most partial refusals were related to individual factors like contact difficulties, relocation, illness, or caregiving demands, as well as apprehension toward procedures like infant blood collection, not disengagement [2,8,14,15]. Similar barriers have been reported in other LMIC birth cohorts, where mobility, informal work schedules, and caregiving demands often hinder consistent follow-up [4,15,16]. Recognizing these as structural, not voluntary, disengagement is essential when interpreting participation patterns and designing adaptive, participant-centered strategies. Moreover, these reasons varied across follow-up waves, shaped by evolving contexts and procedural demands, underscoring the need for flexible retention approaches in rural cohort studies.

The Tracking Planner facilitated real-time updates of participant status, coordination, and allowed early detection of disengagement risks. Weekly reviews by the technical coordinator supported timely adjustments. Flexible scheduling, home visits, and multi-modal interviews were key to maintaining engagement, consistent with evidence from LMIC studies [11,16,17]. Given that all participants were women, caregiving demands, and limited decision-making power likely influenced retention, highlighting the importance of gender-sensitive approaches [18].

Protocol adjustments during pandemic disruptions allowed retention comparisons: 12-month follow-up yielded higher rates than 18-month (84% vs. 75%), supporting evidence that shorter intervals improve retention [2,5]. This flexibility was critical for continuity.

The SEMILLA tracking system aligns with international recommendations for longitudinal research [8,12,19], documenting participation status, reasons for non-completion, and guiding real-time decision-making. Its strengths, including real-time tracking, adaptive coordination, and a five-tier classification, enabled responsive engagement and offer a replicable model for rural LMIC cohorts [1,12,20]. This approach reflects emerging frameworks that advocate for more granular classifications to better capture participation dynamics in longitudinal studies [5,8]. Overall, the system functioned as intended: participation status was updated continuously, risks of attrition were identified early, and adaptive follow-up actions were deployed across waves, enabling sustained engagement despite structural constraints.

Retention was associated with maternal employment and ethnicity. Employed women stayed longer in follow-up, consistent with evidence that stable work supports continuity in longitudinal studies [21]. Indigenous women also showed higher retention than Mestiza women, aligning with studies highlighting how strong community ties foster sustained participation [12]. These findings demonstrate that retention is influenced by operational strategies, social stability, and cultural belonging, providing novel evidence from a rural Latin American cohort.

A key strength of this study is the structured combination and systematic documentation of retention strategies in a rural LMIC context, which distinguishes SEMILLA from other cohorts and provides a replicable model for designing tracking systems in similar settings.

Additionally, structural constraints, including disruptions caused by the COVID-19 pandemic, led to the implementation of two final waves, which offered a valuable opportunity to document retention under different follow-up lengths.

This study has some limitations. Delayed home visits; subgroup analyses were not performed, although they could inform refined retention strategies; it was not possible to isolate the effect of individual components as all participants were exposed to the same tracking system; the partial refusal classification was primarily designed with respect to the main study objectives, and may limit some secondary outcomes by reducing the sample size across waves; finally, while this study focused on documenting the operational design, contextual adaptations, and development of the tracking system, it did not include other implementation outcomes often examined in implementation research, such as acceptability, fidelity, or a full cost analysis.

Future efforts should include budget tracking and dedicated staff to optimize logistics [12,17]. They could also build on this work by assessing these aspects to provide additional evidence for its scalability and transferability to similar tracking systems.

Future research should explore scaling digital tools, using flexible classifications, and engaging communities early to improve long-term retention [19,22,23].

## Conclusion

The SEMILLA experience illustrates how a structured, yet adaptable tracking system can sustain high retention in rural LMIC settings, even amid pandemic disruptions and participant mobility. Key components, including a predefined follow-up schedule, a real-time digital coordination, and a five-tier participation classification, enabled consistent engagement and responsive field operations.

Retention rates and implementation strategies aligned with findings from other LMIC cohorts, such as MINA-Brazil and ISA-Costa Rica, highlighting the value of trained local teams, systematic tracking, and flexible contact protocols. The system also reflected emerging best practices by integrating gender-sensitive planning and granular participation classification. While limitations included delayed implementation of home visits and lack of detailed cost tracking, these did not prevent sustained retention and could inform future improvements.

This experience offers a scalable model emphasizing early planning, adaptable protocols, and local teams to inform retention strategies in global health research in underserved settings through feasible, context-sensitive approaches.

## Supplementary Material

Tables revised.docx

SQUIRE_Checklist_revised_clean.docx

## Data Availability

Once the SEMILLA data have been fully collected, analyzed, and the main findings published, we will consider sharing data with qualified researchers. Given that our study population is vulnerable, particular care must be taken to protect participants’ privacy. Data sharing will be considered under a formal data-sharing agreement, ensuring compliance with ethical guidelines and institutional regulations. This agreement will require users to: (1) utilize the data strictly for predefined research purposes agreed upon in advance, (2) obtain approval from their Institutional Review Board (IRB), (3) ensure secure storage and handling of the data using appropriate technology, (4) acknowledge the data source in any publications and cite the primary investigators, (5) delete or return the data upon completion of the agreed-upon analyses. Requests for data access will be reviewed on a case-by-case basis. Researchers interested in obtaining access should contact the corresponding author for further details.
