# Supplementary material for: Development of a structured tracking system to improve retention in a birth cohort in rural Ecuador
Source: Glob Health Action. 2025 Oct 14;18(1):2569207. doi: 10.1080/16549716.2025.2569207 (PMC12523455; doi:10.1080/16549716.2025.2569207)
Supplement: Tables revised.docx [file ZGHA_A_2569207_SM3873.docx]

**Table 1.** Scheduled follow-up visits by study period and Final Wave

| **Study Period** | **Visit** | **FW1  early enrollment (8–14 wk)** | **FW1**  **late enrollment (15–20 wk)** | **FW2**  **early enrollment (8–14 wk)** | **FW2**  **late enrollment (15–20 wk)** |
| --- | --- | --- | --- | --- | --- |
| Pregnancy | 20 weeks | ✓ | — | ✓ | — |
|  | 32 weeks | ✓ | ✓ | ✓ | ✓ |
| Birth | 0–14 days after birth | ✓ | ✓ | ✓ | ✓ |
| Infancy | 3 months | ✓ | ✓ | ✓ | ✓ |
|  | 6 months | ✓ | ✓ | ✓ | ✓ |
|  | 9 months | ✓ | ✓ | ✓ | ✓ |
|  | 12 months | ✓ | ✓ | ✓ | ✓ |
|  | 15 months | — | — | ✓ | ✓ |
|  | 18 months | — | — | ✓ | ✓ |

Participants were grouped by gestational age at enrollment (8–14 or 15–20 weeks) and assigned to FW1 or FW2. ✓: visit was scheduled for that group; —: not applicable. Collection windows applied to each visit. wk, weeks

**Table 2. Core assessment activities used to classify participant status by study**

**period in the SEMILLA Study**

| **Assessment Activity** | **Study periods** | | |
| --- | --- | --- | --- |
|  | **Pregnancy** | **Birth** | **Infancy** |
| Main questionnaire (sociodemographic, risk factors) | ✓ | ✓ | ✓ |
| Biological samples | Urine, blood (mother) | Blood  (newborn) | — |
| Infant anthropometric  evaluations | — | ✓ | ✓ |
| Infant neurodevelopmental assessments | — | — | ✓ |

**Note**: Core assessment activities were selected based on their alignment with the study’s primary objective, evaluating the relationship between prenatal exposure to ethylene thiourea (ETU) and maternal and infant thyroid function and neurodevelopment. ✓: scheduled in that period; —: not applicable.

**Table 3. Data variables included in the Tracking Planner sheets**

| **Follow-up Planner** | **Participant Contact Data** |
| --- | --- |
| *Participant identifier* | *Participant identifier* |
| *Enrollment date* | *Primary phone number* |
| *Collection windows start/end dates* | *Secondary phone number* |
| *Participant status classification* | *Preferred contact times* |
| *Reason for missed visit* | *Georeferenced address* |
|  | *Comments on contact attempts* |

**Note**: Variables in the follow-up planner and participant contact data sheet.
