# Supplementary material for: Development of a structured tracking system to improve retention in a birth cohort in rural Ecuador
Source: Glob Health Action. 2025 Oct 14;18(1):2569207. doi: 10.1080/16549716.2025.2569207 (PMC12523455; doi:10.1080/16549716.2025.2569207)
Supplement: SQUIRE_Checklist_revised_clean.docx [file ZGHA_A_2569207_SM3872.docx]

**SQUIRE 2.0 Checklist**

This checklist has been completed for the manuscript titled "Development of a Structured Tracking System to Improve Retention in a Birth Cohort in Rural Ecuador", submitted as Methods Forum Article to Global Health Action. The article describes the design, contextual adaptations, and field implementation of a participant tracking system developed within the SEMILLA Study, a community-based birth cohort in Ecuador. While not a formal quality improvement intervention designed to assess clinical outcomes, the manuscript documents operational outcomes on participant retention and data completeness. The structured tracking system represents an embedded approach to strengthen research processes and sustain engagement in a longitudinal health research setting. Aligned with SQUIRE 2.0 reporting principles, it provides methodological and operational insights, including transferable lessons for tracking in resource-constrained settings and contributions to local **capacity building through team training and sustained field expertise**.

| **Title and abstract** | | **Status / Comments** |
| --- | --- | --- |
| 1 | Title | **Completed**  The title clearly reflects the nature of the intervention (a structured tracking system) and its goal (to improve retention) within a rural birth cohort study. Although it does not refer directly to healthcare delivery, retention improvement is a key component of quality and equity in longitudinal health research. By strengthening data completeness and representativeness, retention directly supports the reliability of evidence that informs health policy and practice. This aligns with the broader SQUIRE definition of health improvement. |
| 2 | Abstract | **Completed**  The abstract summarizes all key components of the study, including background, local context (mobility, informal employment, and limited phone access), methods, the intervention (structured tracking system), main operational outcomes related to retention, providing evidence of system performance, and conclusions. While the abstract does not follow a structured format with formal subheadings (Background, Methods, Results, etc.), this is consistent with the journal's guidelines for Methods Forum Articles, which allow for narrative abstracts. The abstract includes relevant indexing terms such as “tracking system”, “retention”, “birth cohort”, “low- and middle-income countries”, and “community-based longitudinal research”, supporting searchability and indexing. |
| **Introduction** | | **Status / Comments** |
| 3 | Problem Description | **Completed**  The manuscript identifies the core problem as the limited documentation and systematization of participant tracking processes in longitudinal cohort studies conducted in rural LMICs. In the SEMILLA Study context, structural barriers— including socioeconomic instability, informal employment, limited phone access, high mobility, institutional mistrust, and limited infrastructure—compromised follow-up. These challenges threaten data quality and risk underrepresenting the most vulnerable populations, ultimately weakening the validity and equity of evidence needed to inform maternal and child health programs in underserved settings. Strengthening participant retention through structured, context-adapted tracking systems supports the quality, efficiency, and fairness of longitudinal research that underpins health decision-making for vulnerable populations. |
| 4 | Available Knowledge | **Completed**  The introduction summarizes existing literature on retention strategies, citing references [1–5]. Reports from high-income countries highlight strategies supported by robust infrastructure, institutional trust, and digital tools. In contrast, evidence from LMICs is fragmented, with most studies documenting isolated practices such as reminder calls or incentives, but rarely describing comprehensive, structured tracking systems adapted to rural or resource-constrained contexts.  Although some foundational references are more than a decade old, they remain relevant given the limited documentation of comprehensive systems in LMICs to date. More recent studies were incorporated where available to complement this evidence. This gap is explicitly noted in the introduction: “Most studies focused on isolated practices, without detailing operational frameworks that can be replicated across multiple waves of follow-up.” |
| 5 | Rationale | **Completed**  The tracking system was developed using a structured approach built around four components. Rather than drawing on a formal theoretical framework, its rationale emerged from practical field needs and contextual knowledge, incorporating participant-centered and flexible strategies. Features such as real-time monitoring, adaptive classifications, and home-based visits were included because they directly addressed the local barriers identified in the SEMILLA Study context. |
| 6 | Specific Aims | **Completed**  The aim of the manuscript is clearly stated: to describe the operational design, contextual adaptations, and implementation experiences of a structured tracking system developed for the SEMILLA Study. The report seeks to share practical lessons and provide guidance for improving participant retention in community-based research in underserved settings. The purpose is to document an operational model that can be replicated or adapted in similar contexts to strengthen research quality. |
|  | **Methods** | **Status / Comments** |
| 7 | Context | **Completed**  This section describes the setting of the SEMILLA Study as a rural area in the Ecuadorian highlands characterized by intensive floriculture, a predominantly female workforce, and high population mobility. It also notes key contextual disruptions—such as the COVID-19 pandemic and national protests—led to a protocol revision creating two final follow-up waves (FW1 and FW2), which maintained common objectives and procedures but differed in duration. This adaptation made SEMILLA unique by allowing retention comparisons across cohorts while reinforcing the need for a flexible, context-adapted tracking system. |
| 8 | Intervention | **Completed**  **8a. Description of the intervention(s):**  This manuscript documents the development of a participant tracking system as an operational strategy, rather than an intervention tested for causal impact. The “Methods” section describes the system’s structure, follow-up schedule, classification process, digital tracking tool, and participant contact procedures in sufficient detail to support adaptation or replication in similar contexts.  **8b. Specifics of the team involved in the work:** The “Methods” section details the composition and responsibilities of the tracking team, including the tracker/recruiter, interviewers, and the technical coordinator. Their roles are described in relation to coordination, data collection, follow-up classification, and operational monitoring. Importantly, the manuscript highlights that the team remained consistent throughout the study period and includes cost estimates of their work, adding transparency to resource requirements and supporting the system’s replicability in other contexts. |
| 9 | Study of the Intervention | ****Completed****  **9a. Approach chosen for assessing the impact of the intervention(s):** This manuscript does not describe a formal intervention designed for causal evaluation, but rather documents the development of a tracking system as an operational process. The “Methods” section describes how operational indicators_—_participant classification status, retention rates, and home visit outcomes—were monitored over time to assess system functioning. In addition, the study analyzed associations between baseline sociodemographic variables and retention status. These analyses provided further insight into which participant characteristics were linked to higher or lower retention, and guide adjustments during fieldwork.  **9b. Approach used to establish whether the observed outcomes were due to the intervention(s):** No causal attribution was attempted, as the aim was to document a field-embedded operational system rather than test an intervention. However, changes in participation patterns, such as improved engagement following the incorporation of home visits, were reviewed qualitatively by the coordination team and complemented by statistical associations with baseline sociodemographic factors. This combined descriptive and analytical approach allowed the team to assess plausibility that observed outcomes were related to the tracking system and to guide iterative adaptations during fieldwork. |
| 10 | Measures | **Completed**  **10a. Measures chosen for studying processes and outcomes of the intervention(s):**  The “Methods” section describes a set of operational indicators used to monitor the tracking system’s functioning, including participant classification status, retention rates, partial refusals, home visit success, and associations between sociodemographic characteristics and retention. These indicators and variables followed standard definitions used in longitudinal cohort research and were applied consistently to support internal monitoring and adaptive management.  **10b. Description of the approach to the ongoing assessment of contextual elements:**  Contextual factors affecting tracking, such as participant mobility, scheduling conflicts, or discomfort with study procedures, were assessed through ongoing field coordination and participant feedback. These assessments informed adjustments such as the formal incorporation of home visits.  **10c. Methods for assessing data completeness and accuracy:**  The “Methods” section explains that the technical coordinator conducted weekly reviews of the Tracking Planner to verify completion of all scheduled follow-up activities and adherence to data collection windows. Discrepancies were discussed and resolved with the field team during coordination meetings to ensure consistency and data quality across waves. |
| 11 | Analysis | **Completed**  **11a. Quantitative and qualitative methods used to draw inferences from the data**  The “Methods” section describes a descriptive analysis of operational data using Excel. Frequencies and proportions were calculated for participant classification categories, retention rates, reasons for partial refusals, and home visit success. In addition, associations between baseline sociodemographic characteristics and retention status were tested using χ², Fisher’s Exact Test, and Student’s t test. No causal modeling was conducted, as the aim was to document patterns in follow-up and explore correlates of retention to inform adaptive management. ****11b. Methods for understanding variation within the data, including the effects of time as a variable**** Variation over time was assessed by examining retention, classification, and repeated partial refusals across follow-up waves. Differences between Final Wave groups (FW1 vs FW2) were analyzed separately to account for differences in cohort duration. These observations provided insight into how engagement evolved over time and informed real-time adaptations to the tracking system**.** |
| 12 | Ethical Considerations | **Completed**  The manuscript explains that the SEMILLA Study received ethical approval from multiple national and international institutions, in accordance with the Declaration of Helsinki and CIOMS 2016 guidelines. The tracking system was implemented with careful attention to participant autonomy and privacy, including documentation of refusals without penalty and flexible procedures to reduce burden. As clarified in the text, home visits were conducted only with participants’ prior verbal agreement, ensuring that follow-up respected autonomy and daily responsibilities. A detailed description of ethical oversight is provided in the “Ethics and consent” section, and no conflicts of interest were declared. |
|  | **Results** | **Status / Comments** |
| 13 | Results | **a. Initial steps of the intervention(s) and their evolution over time (e.g., timeline diagram, flow chart, or table), including modifications made to the intervention during the project**  **Completed** The manuscript describes the development of a structured tracking system, detailing its components (participant classification, home visit strategy, and Tracking Planner). Its evolution is shown through changes in classification patterns and the increasing contribution of home visits across follow-up waves (Figure 3), reflecting adaptations made to sustain participation.  **b. Details of the process measures and outcome**  **Completed**  Process measures include participant classification per wave, frequency and reasons for partial refusals, and the success rate of home visits (88%). Outcome measures included retention rates calculated with two denominators: relative to the total baseline cohort (N=409) and relative to participants in each Final Wave (FW1 and FW2). For example, retention was 84% at 12 months (FW1) and 75% at 18 months (FW2), corresponding to 23% and 54% of the baseline cohort, respectively, and 77% overall. This dual reporting supports comparability and transparency in interpreting retention outcomes. These data are presented in narrative form and figures.  **c. Contextual elements that interacted with the intervention(s)**  **Completed**  The manuscript identifies key contextual challenges affecting follow-up, including the COVID-19 pandemic, national social protests, participant mobility, and individual-level barriers such as illness and scheduling conflicts. These are shown in Figure 4 and discussed under “Partial Refusals.”  **d. Observed associations between outcomes, interventions, and relevant contextual elements**  **Completed**  Beyond operational observations, statistical associations between baseline sociodemographic factors and retention were analyzed. Maternal employment (p=0.017) and self-identified ethnicity (p=0.007) were significantly associated with retention, with Indigenous women showing higher retention (86.4%) compared to Mestiza women (74.8%). These findings demonstrate how contextual and individual factors interacted with retention outcomes and the tracking system.  **e. Unintended consequences such as unexpected benefits, problems, failures, or costs associated with the intervention(s)**  **Addressed**  No major unintended consequences were observed. However, the discomfort expressed by some participants regarding the main questionnaire and neurodevelopmental evaluations is acknowledged. The estimated cost of home visits (USD 2,500) is also reported. This figure includes only transportation expenses, as there was no dedicated system for recording other operational costs such as phone calls or field time invested during home visit coordination. In addition, resource requirements for the tracking team were documented in the Methods section to provide transparency on staffing costs throughout the study.  **f. Details about missing data**  **Completed**  Missing data were addressed through the tracking system’s classification categories (partial refusal, dropped out). These designations were used to calculate retention rates and monitor field performance. No statistical imputation was conducted. |
|  | **Discussion** | **Status / Comments** |
| 14 | Summary | **a. Summary of key findings**  **Completed**  The discussion highlights that the tracking system sustained high retention rates (84% at 12 months, 75% at 18 months), despite contextual challenges. It summarizes the key contributors and associations with retention, and includes comparisons between Final Waves, illustrating the effect of follow-up duration.  **b. Particular strengths of the project**  **Completed**  Strengths include the use of a flexible, real-time Tracking Planner; a well-trained and consistent field team; and the introduction of a nuanced five-category classification system including “partial refusal.” These elements enhanced data quality, retention, and internal monitoring. The SEMILLA experience provides a systematically documented and replicable model for rural LMIC cohorts. |
| 15 | Interpretation | **a. Nature of the association between the intervention(s) and the outcomes**  **Completed** The tracking system was not evaluated as a formal intervention, but retention outcomes were described in relation to implementation processes. Home visits were associated with improved engagement of otherwise unreachable participants, and statistical analyses showed significant associations between retention, maternal employment, and ethnicity.  **b. Comparison of results with findings from other publications**  **Completed**  Findings are compared to Latin American cohorts (e.g., MINA-Brazil, ISA-Costa Rica) and to other LMIC experiences (e.g., Vientiane, Lao PDR), aligning with global literature supporting participant-centered, adaptive tracking. The discussion also notes that the SEMILLA system contributes to emerging frameworks recommending more granular classifications to capture participation dynamics.  **c. Impact of the project on people and systems**  **Completed**  The tracking system functioned as a core operational structure, enabling real-time decision-making and participant engagement. It also strengthened local capacity by sustaining a trained team and reinforcing skills in participant follow-up, data quality assurance, and adaptive study management.  **d. Reasons for differences between observed and anticipated outcomes, including the influence of context**  **Completed**  The discussion acknowledges that 12-month retention was higher than at 18 months, possibly due to shorter follow-up intervals. It also explains how external factors (e.g., pandemic, social unrest) led to protocol modifications and influenced retention patterns.  **e. Costs and strategic trade-offs, including opportunity costs**  **Addressed**  While transportation costs for home visits were estimated (~USD 2,500), the absence of a dedicated cost-tracking system limited a comprehensive assessment of expenses. The need for better resource planning and budget monitoring is noted as a key recommendation. |
| 16 | Limitations | **Completed**  The discussion addresses operational limitations. Internal validity is limited by the inability to isolate the effect of individual tracking components, the absence of subgroup analyses, and the design of the “partial refusal” category, which may reduce sample size for certain secondary analyses. In addition, the study did not include other operational outcomes such as acceptability, fidelity, or a full cost analysis. Efforts were made to minimize limitations through real-time monitoring, weekly reviews of the Tracking Planner to ensure data completeness, and the incorporation of home visits to reduce loss to follow-up. |
| 17 | Conclusions | **Usefulness, sustainability, and potential for spread of the work**  **Completed**  The manuscript concludes that the SEMILLA experience illustrates how a structured but adaptable tracking system can sustain high retention in rural LMIC settings, even amid pandemic disruptions and participant mobility. Usefulness is shown through predefined schedules, real-time coordination, and a five-tier classification that enabled consistent engagement and responsive field operations. Sustainability was supported by trained local teams and flexible protocols, and the model offers potential for spread to other LMIC cohorts through feasible, context-sensitive approaches. Implications for practice include the value of early planning, gender-sensitive adaptations, and granular classifications to improve retention strategies. Suggested next steps include strengthening cost-tracking mechanisms and integrating operational refinements such as earlier incorporation of home visits. |
|  | **Others** | **Status/Comment** |
| 18 | Funding | **Completed**  Funding source declared in Declarations. |

**Note:** Items referring to “intervention” were interpreted in the context of a field-embedded operational strategy. The participant tracking system was not a formal intervention tested for causal effect, but a process developed and implemented to strengthen retention in a community-based cohort study. All references to “intervention” should be understood accordingly.
